# Assessment of Dynamic Change of Coronary Artery Geometry and Its Relationship to Coronary Artery Disease, Based on Coronary CT Angiography

**DOI:** 10.1007/s10278-019-00300-5

**Published:** 2019-11-19

**Authors:** Jordy K. van Zandwijk, Volkan Tuncay, Rozemarijn Vliegenthart, Gert Jan Pelgrim, Cornelis H. Slump, Matthijs Oudkerk, Peter M. A. van Ooijen

**Affiliations:** 1 University Medical Center Groningen, University of Groningen, Groningen, The Netherlands; 2 MIRA Institute for Biomedical Technology and Technical Medicine, University of Twente, Enschede, The Netherlands; 3 Department of Radiology, University Medical Center Groningen, University of Groningen, PO Box 30001, NL-9700 RB Groningen, The Netherlands; 4 Department of Radiation Oncology, University Medical Center Groningen, University of Groningen, Groningen, The Netherlands

**Keywords:** CT angiography, Coronary artery geometry, Curvature, Tortuosity, Coronary arteries, Coronary artery disease

## Abstract

To investigate the relationship between dynamic changes of coronary artery geometry and coronary artery disease (CAD) using computed tomography (CT). Seventy-one patients underwent coronary CT angiography with retrospective electrocardiographic gating. End-systolic (ES) and end-diastolic (ED) phases were automatically determined by dedicated software. Centerlines were extracted for the right and left coronary artery. Differences between ES and ED curvature and tortuosity were determined. Associations of change in geometrical parameters with plaque types and degree of stenosis were investigated using linear mixed models. The differences in number of inflection points were analyzed using Wilcoxon signed-rank tests. Tests were done on artery and segment level. One hundred thirty-seven arteries (64.3%) and 456 (71.4%) segments were included. Curvature was significantly higher in ES than in ED phase for arteries (*p* = 0.002) and segments (*p* < 0.001). The difference was significant only at segment level for tortuosity (*p* = 0.005). Number of inflection points was significantly higher in ES phase on both artery and segment level (*p* < 0.001). No significant relationships were found between degree of stenosis and plaque types and dynamic change in geometrical parameters. Non-invasive imaging by cardiac CT can quantify change in geometrical parameters of the coronary arteries during the cardiac cycle. Dynamic change of vessel geometry through the cardiac cycle was not found to be related to the presence of CAD.

## Introduction

Coronary artery disease (CAD) is the most common type of heart disease and the leading cause of death worldwide [1]. Hemodynamics and geometry of the coronary artery have been suggested to play a role in the development of atherosclerotic plaque, but these relationships are complex and still matter of debate [2]. Previous hemodynamic studies using flow simulations in static vessel models suggest an association between low wall shear stress and coronary plaque development. Thus, there may be preferred sites for plaque development depending on mechanical factors [2–6]. In studies on coronary hemodynamics in simulations incorporating cardiac motion, the importance of considering physiologically realistic flow and vessel motion was stressed [7–11]. This implies the need for *in vivo* patient studies.

So far, the only evidence regarding dynamic changes of the coronary geometry originates from invasive coronary angiography (ICA). ICA is still considered the reference standard for the diagnosis of CAD, but is limited by its invasiveness and its lack of information about plaque characteristics [12]. There are scarce data from ICA studies about coronary geometry and the relation with CAD related events. Zhu *et al.* classified human coronary geometry in a single, diastolic phase [13]. O’Loughlin *et al*. found that the ratio of segment length between systolic and diastolic phase can predict the location of future culprit lesions causing myocardial infarction [14]. Coronary computed tomography angiography (cCTA) is a non-invasive imaging technique that is nowadays an accepted alternative in coronary artery evaluation [15]. cCTA is safer and cheaper than ICA, and associated with less discomfort to the patient. Three-dimensional change in coronary artery geometry during the cardiac cycle and its relationship to CAD can be investigated using cCTA with electrocardiographically synchronized data acquisition during systolic and diastolic phase. A previous study correlated the ratio of coronary artery length between end-systolic (ES) and end-diastolic (ED) phases with the location of atherosclerotic lesions, based on dual-source CT [16]. Only information about the length of coronary segments was obtained, rather than specific geometric information. Recent dedicated software packages now enable the extraction of quantifiable three-dimensional parameters of vessel geometry, derived from multiple phases of the cardiac cycle in cCTA. In a recent cCTA-derived cross-sectional study, a relationship between measures of curvature and tortuosity, and the presence and extent of CAD was found in a single cardiac phase [17]. Whether dynamic change of coronary curvature and tortuosity through the cardiac cycle affects the relationship with CAD is unknown.

The current study focuses on the assessment of coronary artery geometry through the cardiac cycle based on cCTA, and the association of dynamic change in coronary artery geometry with CAD. Our hypotheses are (1) coronary artery geometry changes dynamically during the cardiac cycle; (2) the degree of stenosis and plaque types are related to dynamic changes in geometrical parameters. These hypotheses are based on previous recommendations indicating to look more closely towards the relation of dynamic coronary artery geometry with the coronary lesion location [14, 17]. The novelty of this research resides in its uniqueness to quantify characteristics of movement of the coronary arteries during the cardiac cycle in a non-invasive and three-dimensional way, and in the derivation of new quantitative imaging biomarkers based on cCTA scans. This study did not investigate the relationship between the absolute coronary artery geometry values and CAD as this relationship has already been assessed previously [17–19]. The current study is exploratory. In a previous study by Tuncay et al., coronary geometry measures based on a single (diastolic) phase of the cardiac cycle were found to be related to the presence of CAD. While the assumption is that dynamic changes through the ECG cycle are related to mechanical forces that affect development of plaque, this has been very difficult to study so far due to the lack of a non-invasive method. In this study, we have first of all evaluated the CT-based geometric changes through the cardiac cycle, and assessed whether the size of geometric changes is related to plaque presence, as a potential first hint to a non-invasive, explanatory marker for preferential sites for plaque development.

## Materials and Methods

### Patients

Data from patients involved in different scientific studies from April 2006 until April 2007 were included in this retrospective analysis [20–22]. Patients had either a high probability of CAD [20], were planned for elective conventional invasive coronary angiography (ICA) [21], or were assessed at the emergency department because of acute chest pain [22]. Patients were excluded if they had previous heart surgery or percutaneous coronary intervention (PCI), or if they had a coronary anomaly on cCTA. cCTA was performed at a single tertiary center. Approval from the Medical Ethical committee was originally obtained for each scientific study, and informed consent was obtained from all patients at the time of inclusion. For the current analysis, the Medical Ethical committee waived informed consent requirement because of the retrospective nature of this study without additional burden to the patients involved.

### Coronary CT Angiography Scan Protocol

cCTA was performed on a first-generation dual-source CT system (SOMATOM Definition, Siemens, Erlangen, Germany) using a standardized protocol. The standard scanning protocol involved spiral scanning at 120 kV with retrospective ECG gating. Patients were administered intravenous beta-blockers (metoprolol, 5–20 mg) if the heart rate was above 65 beats per minute, unless in case of contraindications to beta-blockers. All patients were given sublingual nitroglycerin (0.4 mg) prior to the scan protocol. Table pitch was dependent on the heart rate, with a cranio-caudal scan direction starting above the coronary ostia and ending below all cardiac structures at the diaphragm. Contrast-enhanced scan acquisitions were made with a non-ionic contrast agent (Iomeprol 400 mg I/ml, Iomeron® 400, Bracco, Italy) with contrast volume and infusion rate individually determined for each patient. Piers et al. [21] described the scan protocol in more detail. The acquired data were 64 × 2 × 0.6 mm, reconstructed with an increment of 0.4 mm, for coronary analysis. The mean dose length product (DLP) was 1323 ± 288 mGy cm (22.5 ± 4.9 mSv). To be included for the current analysis, reconstructions needed to have been made at every 10% of the R-R interval, with overlapping reconstructed slice thickness of 2.0 mm based on 64 × 0.6 mm slice collimation (originally reconstructed for functional analysis).

### Coronary Artery Assessment

Radiologists with experience in cardiac CT ranging from 5 to over 10 years evaluated the cCTA data for the presence and severity of CAD as part of the research projects. Coronary evaluations were performed using dedicated advanced visualization software (Syngo, Siemens, Erlangen, Germany). The presence of plaque, plaque type (calcified, non-calcified, and mixed) and degree of stenosis were assessed per segment, according to the 15-segment modified American Heart Association (AHA) classification [23].

### Coronary Artery Geometry Assessment

Reconstructed cCTA data were loaded onto a dedicated workstation (Aquarius iNtuition, Ver.4.4.11, TeraRecon, San Mateo, USA). A built-in threshold-based left ventricular ejection fraction (LVEF) analysis function automatically determined the ES and ED phase of the cardiac cycle based on respectively the minimum and maximum filling of the left ventricle. For the current study, in case the reconstruction quality of the coronary arteries in the automatically determined ES or ED phase was too low, another phase with diagnostic depiction of the coronary arteries was selected up to two phases shifted from the minimum or maximum filling. If optimal quality could not be obtained based on these restrictions, the coronary artery was excluded from further analysis.

The resulting ES and ED phases were used for detailed inspection of the coronary arteries and its individual segments, according to the 15-segment modified AHA classification [23]. For the right coronary artery (RCA), we assessed the proximal, mid, and distal segment. For the left coronary artery, we assessed the left main artery, proximal, mid, and distal segment of the left anterior descending (LAD) artery, and the proximal and distal segment of the left circumflex (LCx) artery. On artery level, the RCA, LAD, and LCx were assessed separately. In cases where no side branches were present to identify the end of the segment, we maintained equal pre-set lengths in both ES and ED phase to make sure comparable parts of the vessel were considered. Segments were terminated or excluded when they were smaller than 1.5 mm in average diameter, had a bad reconstruction quality due to the presence of artefacts or incorrect centerline extraction, or were not visible at all. Arteries were excluded if one or more segments could not be assessed.

**Fig. 1:**
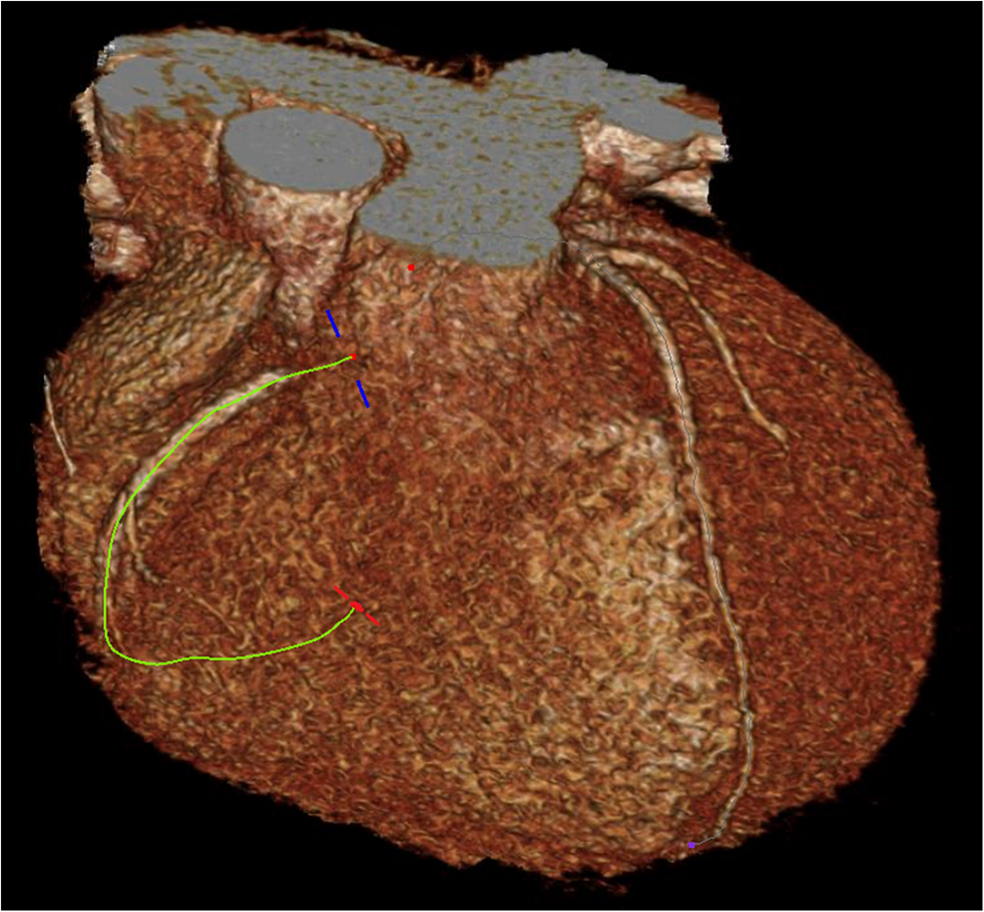
Example of a volume rendered 3D image with centerline extractions of the RCA (selected) and LAD at 70% of the R-R interval (ED phase)

The coronary arteries were selected on the three-dimensional volume-rendered reconstruction (see Fig. 1) or transverse slices in the relevant phase in order to initiate automatic centerline extraction of an artery. The curved planar reformation (CPR) view was reconstructed based on this centerline. CPR is merely a method of visualization of the data that makes it easier to perform the relevant measurements. The centerline that the software automatically created did not always perfectly follow the center of the lumen of the vessel over the entire trajectory. Where the automatic centerline was not in the middle of the vessel lumen, this was manually adapted. The manual adaption was carefully conducted in case the automatic centerline creation deviated from the actual centerline. Markers were applied at the beginning and endpoint of a segment. Measurements were performed for each segment and each entire artery.

The centerline can be described as a parametrically defined space curve in three dimensions in Cartesian coordinates given by *γ*(*t*) = (*x*(*t*), *y*(*t*), *z*(*t*)), with *t* ranging from 1 to *n* points of the centerline. The following geometric parameters were assessed: mean curvature (*κ* in mm^−1^, see Eq. 1), tortuosity (*T*, see Eq. 2), and number of inflection points.

1 $$ \kappa ={\sum}_{i=1}^n\frac{4{A}_i}{\left|\gamma \left({t}_i-s\right)-\gamma \left({t}_i\right)\right|\ \left|\gamma \left({t}_i\right)-\gamma \left({t}_i+s\right)\right|\ \left|\gamma \left({t}_i+s\right)-\gamma \left({t}_i-s\right)\right|} $$

2 $$ T=\frac{\sum_{i=1}^{n-1}\sqrt{{\left(x\left({t}_i\right)-x\left({t}_{i+1}\right)\right)}^2+{\left(y\left({t}_i\right)-y\left({t}_{i+1}\right)\right)}^2+{\left(z\left({t}_i\right)-z\left({t}_{i+1}\right)\right)}^2}}{\sqrt{{\left(x\left({t}_1\right)-x\left({t}_n\right)\right)}^2+{\left(y\left({t}_1\right)-y\left({t}_n\right)\right)}^2+{\left(z\left({t}_1\right)-z\left({t}_n\right)\right)}^2}} $$

**Fig. 2:**
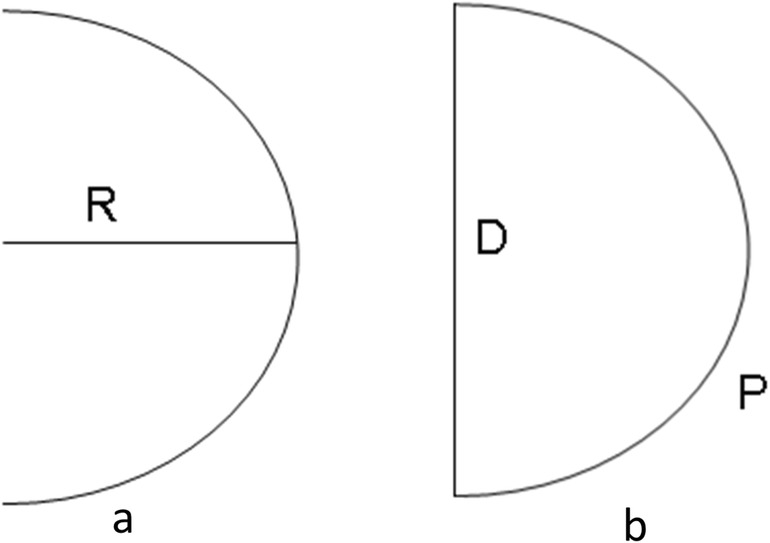
Curvature (**a**) was defined as inverse radius (R) and tortuosity (**b**) was defined as path length (P) divided by strait distance (D)

**Fig. 3:**
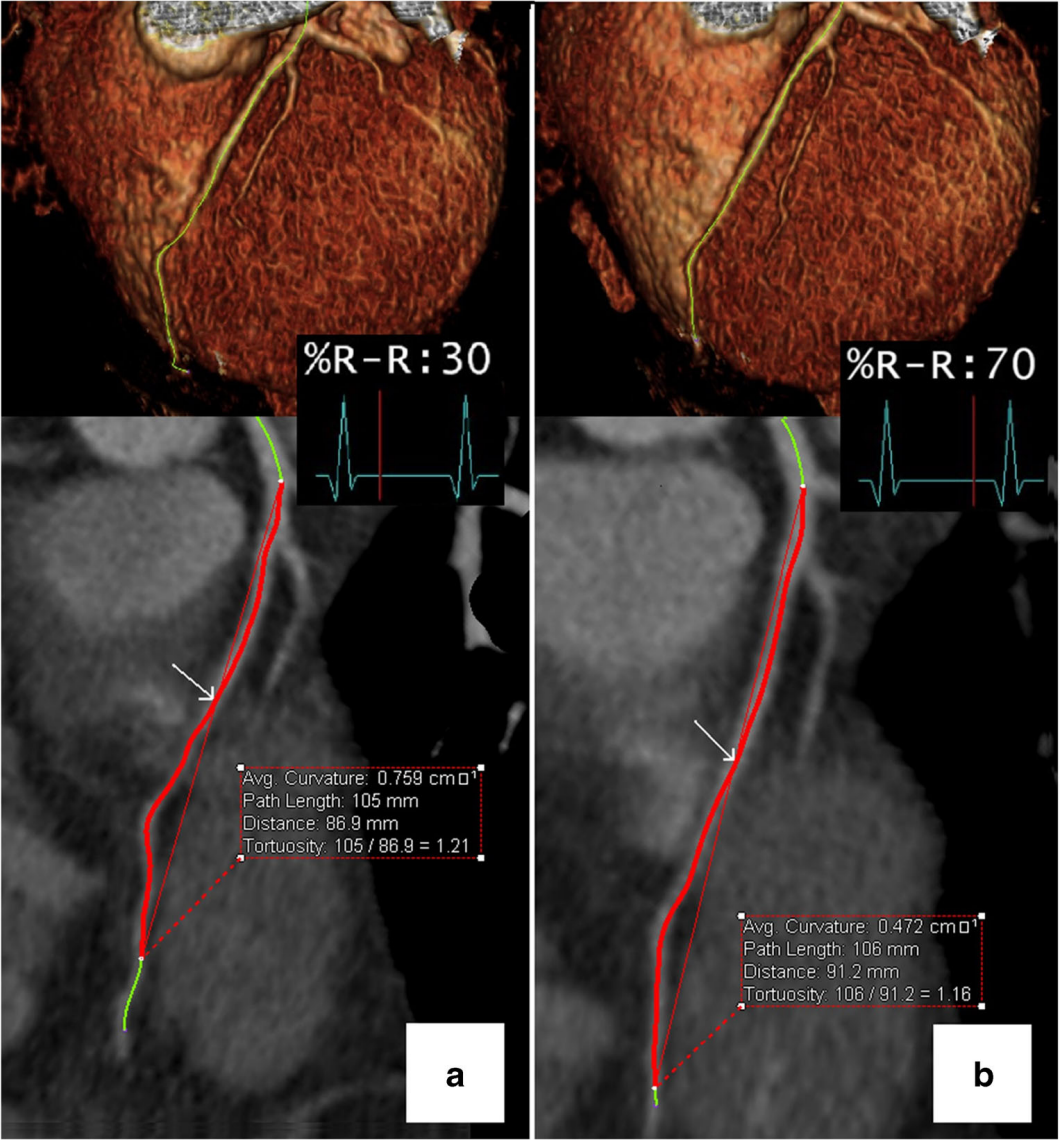
Geometrical measurements of the LAD at 30% in ES phase (**a**) and at 70% in ED phase (**b**). According to these measurements, the LAD has 26% higher curvature, and 3% higher tortuosity in ES phase. The white arrows indicate one inflection point in both phases

Local curvature was defined as reverse radius (Fig. 2a) and calculated using Menger’s formula (Eq. 1), at every 5 mm. The average of the local curvature for the selected path is given in mm^−1^ as the final result for curvature. The ratio of the total path length to the straight distance between begin and endpoint of the indicated range was calculated to determine the tortuosity (Fig. 2b) (Eq. 2). The number of inflection points is determined by the maximum number of intersections (inflection points) that the straight connection between beginning and endpoint has with the centerline when rotating the CPR view. Figure 3 depicts a patient example of geometric parameters in the ES and ED phase.

### Statistics

Normality of the data was assessed using Shapiro-Wilk analysis. Based on the available sample size of arteries and segments, data were considered to have a normal distribution when the test statistic was greater than 0.9. Curvature, tortuosity, and number of inflection points were assessed in the ES and ED phase. For curvature and tortuosity, differences were calculated as the value in ES phase minus the value in ED phase. For number of inflection points, absolute differences were assessed. The differences between ES and ED phase curvature and tortuosity measurements were tested with linear mixed model. The results were corrected for segment and artery information. On the other hand, the differences between number inflection points were tested with the non-parametric Wilcoxon signed-rank test.

Linear mixed models were applied to investigate associations between change in geometrical parameters and the severity of CAD. Severity of CAD was categorized into a number of dichotomous variables: no plaques and plaques with no lumen narrowing (LN negative group) versus plaques with lumen narrowing (LN positive, this group includes all degrees of narrowing), plaques with < 50% stenosis versus plaques with > 50% stenosis, and plaques with < 70% stenosis versus plaques with > 70% stenosis.

Associations of change in geometrical parameters with plaque types (calcified, non-calcified, and mixed) were investigated using linear mixed models. Estimated marginal means were used between groups in the linear mixed model depicting lumen narrowing, stenosis or plaque type.

All statistical analyses were performed in IBM SPSS Statistics version 22.0.0.1 (SPSS Inc, Chicago, USA). Significance for difference was expressed with *p* values, where a two-tailed *p* value of < 0.05 was considered significant.

## Results

Seventy-one patients in whom at least one artery could be assessed were included in this study. Mean age was 62.2 ± 9.9 years, and 87.3% were men (Table 1). The heart rates of four patients were not recorded. Mean heart rate of the remaining 67 patients was 63.7 ± 11.4 beats per minute. In total, 213 arteries and 639 segments were assessed, of which 137 arteries (64.3%) and 456 segments (71.4%) could be included. Arteries and segments that could not be assessed either did not have sufficient quality in both phases, or were too small. The group of arteries consisted of 53 RCA (38.7%), 45 LAD (32.8%), and 39 LCx arteries (28.5%). In total, 114 arteries (83.2%) and 270 segments (59.2%) contained plaque. 0%, < 50%, 50–70%, and > 70% stenosis were observed in 5 (3.6%), 42 (30.7%), 18 (13.1%), and 49 (35.8%) of the arteries respectively. On segment level, 0%, <50%, 50-70%, and > 70% stenosis were observed in 51 (11.2%), 101 (22.1%), 43 (9.4%), and 75 (16.4%) of the segments respectively.

**Table 1:** Clinical characteristics of the study population

	All patients (*N =* 71)
Age (years)	62.2 ± 9.9
Male gender (%)	87.3
Body mass index (kg/m^2^)^a^	27.4 ± 3.7
Hypertension (%)^b^	55.7
Dyslipidemia (%)^c^	63.5
Diabetes mellitus (%)^c^	23.8
Smoking (%)^d^	54.8

Continuous variables are expressed as mean ± standard deviation or median (25th, 75th percentile), dichotomous variables are expressed as percentages

^a^Information was available for 37 patients; *LDL* low density lipoprotein

^b^Information was available for 61 patients

^c^Information was available for 63 patients

^d^Information was available for 62 patients

In systole, the segments were most frequently best assessable at 40% of the R-R interval (*n* = 227, 49.8%, range 5–60%). In 11.6% of the segments, deviation was needed from the original, software-indicated ES phase due to low reconstruction quality or motion artefacts. In diastole, the segments were most frequently best assessable at 90% of the R-R interval (*n* = 172, 37.7%, range 70–110%). In 66.9% of the cases, we deviated from the original ED phase. Although the assessed cases at 110% (i.e., 10%) of the R-R interval (*n* = 41, 9.0%) are strictly part of the subsequent cardiac cycle, they were found to have a sufficient filling of the left ventricle to be assessed as diastolic phase.

**Fig. 4:**
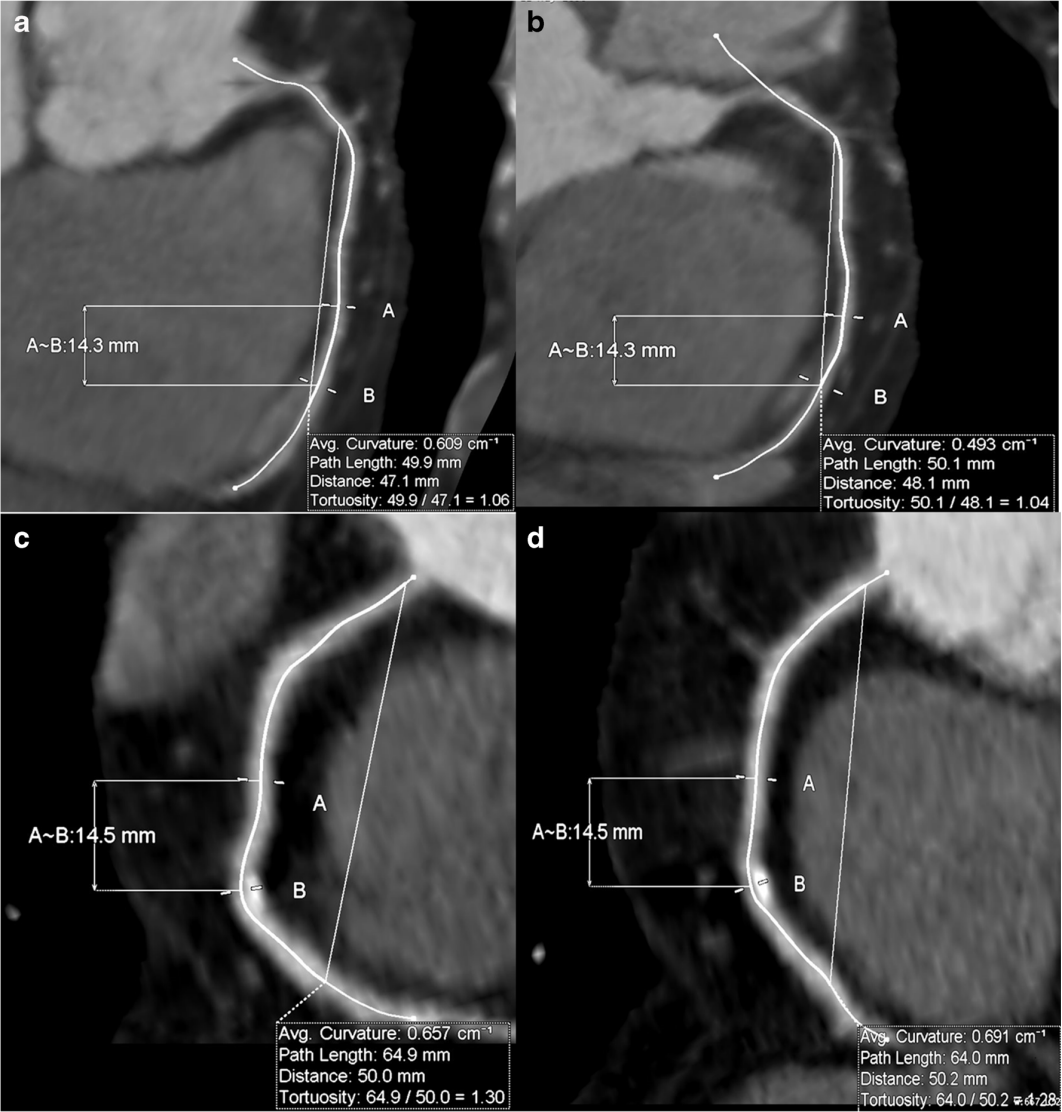
Curvature and tortuosity measurements of a patient with no significant stenosis at 30% in ES phase (**a**) and at 70% in ED phase (**b**); and a patient with no significant stenosis at 30% in ES phase (**c**) and at 80% in ED phase (**d**)

Table 2 shows overall values for curvature, and tortuosity in ES and ED phase on (individual) artery and segment level. Figure 4 shows the sample measurement results of a patient without significant stenosis (panels a and b) and a patient with stenosis (panels c and d).

**Table 2:** Outcomes of the measured parameters in end-systolic (ES) and end-diastolic (ED) phase on artery and segment level, depicted as mean (mean standard error). Significant differences are indicated with an asterisk

		Arteries (*n* = 137)		Segment (*n* = 456)	
Parameter	Phase	Outcome	*p* value	Outcome	*p* value
Curvature (mm^−1^)	ES	0.090 (0.002)	0.002*	0.085 (0.002)	< 0.001*
	ED	0.081 (0.002)		0.078 (0.001)	
Tortuosity	ES	1.36 (0.026)	0.09	1.12 (0.005)	0.005*
	ED	1.33 (0.026		1.10 (0.005)	
Inflection points	ES	2.20 (0.095)	< 0.001*	0.63 (0.038)	< 0.001*
	ED	1.96 (0.091)		0.51 (0.034)

### Curvature

Curvature in ES and ED phase and differences in curvature were normally distributed. Compared to the ED phase, the mean curvature was 11.1% higher in the ES phase on artery level (*p* = 0.002), and 8.9% higher on segment level (*p* < 0.001) (Table 2).

### Tortuosity

Tortuosity differences were normally distributed. The mean tortuosity was 2.3% higher in end-systole than in end-diastole on artery level (*p* = 0.09), and 1.8% higher on segment level (*p* = 0.005) (Table 2).

### Inflection Points

The difference in number of inflection points was not normally distributed on artery and on segment level (Shapiro-Wilk statistic value 0.755 and 0.564, respectively (both *p* < 0.001)). The mean number of inflection points was significantly higher at ES phase than at ED phase for both artery (*Z* = 3.793, *p* < 0.001) and segment level (*Z* = 5.415, *p* < 0.001) (Table 2).

### Associations Between Geometrical Parameters and Stenosis

There was no significant association between the change in geometric parameters through the cardiac cycle, and stenosis (Tables 3 and 4).

**Table 3:** Linear mixed model associations between geometrical parameters and stenosis on artery level. Dependent variables (differences between ES and ED values) are depicted in the rows, factors in columns. LN− means group with no lumen narrowing, LN+ the group segments with lumen narrowing. EMM are estimated marginal means, with in parenthesis the standard errors

Change in geometrical parameters (∆)	LN−	LN+	*p* value	Stenois < 50%	Stenosis > 50%	*p* value	Stenonis < 70%	Stenosis > 70%	*p* value
Curvature (mm^−1^) EMM	0.006 (0.003)	0.008 (0.001)	0.428	0.006 (0.002)	0.009 (0.002)	0.142	0.006 (0.001)	0.009 (0.002)	0.241
Tortuosity EMM	0.057 (0.012)	0.036 (0.006)	0.098	0.035 (0.007)	0.046 (0.008)	0.299	0.039 (0.007)	0.042 (0.009)	0.785
Inflection points EMM	0.11 (0.13)	0.27 (0.07)	0.281	0.16 (0.08)	.031 (0.08)	0.185	0.24 (0.07)	0.23 (0.10)	0.913

**Table 4:** Linear mixed model associations between geometrical parameters and stenosis on segment level. Dependent variables (differences between ES and ED values) are depicted in the rows, factors in columns. LN− means group with no lumen narrowing, LN+ the group segments with lumen narrowing. EMM are estimated marginal means, with in parenthesis the standard errors

Change in geometrical parameters (∆)	LN−	LN+	*p* value	< 50%	> 50%	*p* value
Curvature (mm^−1^) EMM	0.006 (0.001)	0.008 (0.001)	0.279	0.007 (0.001)	0.008 (0.002)	0.607
Tortuosity EMM	0.019 (0.003)	0.016 (0.003)	0.440	0.018 (0.002)	0.017 (0.004)	0.798
Inflation points EMM	0.14 (.003)	0.10 (0.03)	0.339	0.13 (.003)	0.10 (0.04)	0.624

### Associations Between Geometrical Parameters and Plaque Types

Association results for plaque type are given in Table 5. None of the dynamic geometrical parameters were significantly associated with type of plaque.

**Table 5:** Linear mixed model associations between geometrical parameters and plaque type at segment level. EMM are estimated marginal means, with in parenthesis the standard errors

Change in geometrical parameters (∆)	Calcified	Non-calcified	Mixed	*p* value
Curvature (mm^−1^) EMM	0.007 (0.002)	0.005 (0.003)	0.010 (0.002)	0.427
Tortuosity EMM	0.018 (0.004)	0.015 (0.006)	0.020 (0.004)	0.796
Inflation points EMM	0.17 (0.05)	0.11 (0.07)	0.06 (0.05)	0.317

## Discussion

This study shows that the geometric parameters of coronary arteries change significantly between the ES and ED phase except for the tortuosity at the artery level, which means the first hypothesis that the coronary artery geometry changes dynamically during the cardiac cycle is accepted. However, based on the results of the statistical analyses, the second hypothesis, which was the degree of stenosis and plaque types are related to dynamic changes in geometrical parameters, was rejected, since we found no significant relationship between geometric changes through the cardiac cycle and extent of CAD*.* To our knowledge, this is the first patient study quantifying coronary geometry during the cardiac cycle based on straightforward post-processing of four-dimensional non-invasive imaging data.

Previous studies focusing on the relation between vessel geometry, corresponding hemodynamics [7–9, 11], and plaque development [6, 18, 19, 24] were often based on computational fluid dynamics and were thus accompanied by modeling assumptions requiring a considerable amount of time and computational capacity [6, 25]. These studies found that hemodynamic and geometric parameters can be linked to (early) plaque development, but conclusions were drawn based on modeling or static approaches of the coronary arteries. Moreover, there have been studies quantifying and cataloguing the geometric parameters of coronary arteries with invasive imaging techniques. Zhu *et al*. used ICA to measure geometry of the proximal, mid, and distal segments separately of the RCA and LAD in one phase and found, for instance, a twofold difference in maximum curvature in LAD segments between individuals. They state that a particular geometric feature must exhibit large variability between vascular regions or individuals, if it should be considered as an atherosclerotic risk factor. They also suggested to add a dynamic dimension in addition to their work, which can create even more variability [13]. In the study of Johnson et al., biplane cine angiography was used for the quantifications, which is an invasive method in contrast to CT, and so only applicable in selected patients at high suspicion of CAD. Furthermore, this involves two projection directions, requiring additional reconstruction algorithms to obtain three-dimensional information [26]. In a recent study, Tuncay et al. assessed the relationship between curvature and tortuosity of coronary arteries and the presence of significant stenosis and plaque using cCTA [17]. They reported that static coronary artery geometry is related with the presence of plaque and significant stenosis. In this study, all the analyses were done in diastolic phase. The effect of dynamic change of coronary artery geometry was not investigated. The only patient studies slightly resembling the current study in terms of dynamic changes through the cardiac cycle in cCTA were conducted by O’Loughlin et al. [16] and Griffiths et al. [27]. Both studies were conducted by same study group. In the study of O’Loughlin et al., authors only investigated the relationship between the index they called quantitative coronary artery motion (QCAM) which was defined as ((centerline length for section X at end-systole)/(centerline length for section X at end-diastole)%) and location of plaques. They only included a few segments of the coronary tree of 25 patients. The authors observed significant correlation between plaque location and QCAM (*p* = 0.023)). In the study of Griffiths et al., they further investigated if change of tortuosity is related to the location of plaques with 14 patients. They could not observe significant relationship between the dynamic change of tortuosity and location of plaques. Strength of our study resides in the fact that coronary arteries were geometrically quantified based on four-dimensional cCTA data involving multiple phases of the cardiac cycle. Our method involves more detailed and three-dimensional geometric information in terms of curvature, tortuosity, and inflection of both segments and arteries, through the cardiac cycle.

Our method of describing the coronary geometry was mainly based on the curvature and tortuosity parameters derived from a spatial representation of the vessel (i.e., vessel centerline or vessel medial line). Tortuosity in coronary arteries has previously been assessed based on the number of bends in the vessel [5, 28–30]. The measurements of Li et al. for example are therefore substantially differing from ours, resulting in a more tortuous LAD than RCA based on their tortuosity definition [29]. We implemented tortuosity as ratio of path length (L) divided by the straight distance (C), which has also been applied in larger blood vessels [31–33]. Furthermore, curvature calculation was implemented as departure from straightness, using the extracted centerline as a polynomial fitted curve in multiple studies [4, 8, 13, 31, 34, 35]. Our method averages the Menger’s curvature with a 5 mm scale between points, which is an adequate and robust option in order to not significantly affect our results or limit the accuracy of our method.

The inflection points that were measured are a more semi-quantitative evaluation, indicating that certain segments and arteries (especially the LAD) are more compressed in the ES phase than in the ED phase. An equivalent parameter has only twice been introduced for femoral [36] and intracerebral vasculature [37], but has never been applied for coronary arteries or linked with plaque development. However, compression of coronary arteries may be an indicator for sites of lower wall shear stress [5]. Therefore, this novel introduced parameter may help in predicting future sites of CAD.

Previously, it was reported that coronary artery curvature and tortuosity at diastolic phase are significantly related to presence and extend of CAD. However, in the current study, dynamic change of coronary artery geometry metrics curvature, tortuosity, and inflection points were not related to the presence and extent of CAD. This might be due to the fact that the subject population was composed of a selected population of either patients with high probability of CAD or with acute chest pain, resulting in relatively high prevalence of CAD and limiting the range of disease. Furthermore, Griffiths et al. also did not find an association between tortuosity and CAD. They could only associate their QCAM metric based on the length of the specified section of artery to CAD [27]. The results of the studies combined suggest that perhaps overall coronary geometry (whether in systole or diastole) is more important factor related to atherosclerosis than the change during the cardiac cycle. A future study with a large patient population containing substantial number of control subjects can further explore which of the dynamic coronary artery geometry metrics are related to CAD.

This study has some limitations. Firstly, we performed measurements based on a semi-automatically extracted centerline in the CT datasets. The centerline creation did not always yield an accurate result. Especially extraction of severely diseased coronary arteries was frequently incorrect, thus requiring manual adaptation of the centerline to achieve a better match with the vessel trajectory. This could have affected our geometric measurements. Secondly, corresponding segments from the same patient were equivalently adapted in both phases to minimize intra- and inter-observer variability, but this is still a possible shortcoming of the study since variability was introduced. Furthermore, in case image quality at the automatically determined ES or ED phase was too low, another phase was selected up to two phases from the minimum or maximum filling. For example, the segments were most frequently best assessable at 90% of the R-R interval. However, this was only possible in the 37.7% of the cases. In 62.3% of the cases, the coronary arteries were not assessable in the 90% phase. This might also compromise the assessment of dynamic geometry change of coronary arteries between ES and ED phase. Moreover, this is a retrospective non-randomized study. Same quantification technique can be employed in a randomized prospective study with a larger patient group in order to further investigate the relationship between dynamic behavior of coronary arteries and CAD. Future studies should also investigate the influence of some factors that could not be included in this study such as gender differences; the number, caliber, and position of collaterals; and presence of myocardial bridging on the dynamic behavior of coronary artery geometry. In addition to these limitations, high prevalence of obstructive CAD in the population can be counted as one of the limitations. Finally, this study was performed using older dual-source CT technology, which at the time involved retrospective ECG gating for cCTA, resulting in high, nowadays outdated, radiation dose. We do not suggest to perform four-dimensional CT imaging of the coronary arteries for clinical routine, but merely used the existing data from previous studies to extract additional information which allowed to test our hypothesis on geometrical changes through the cardiac cycle, and its relation to CAD. However, with newer CT technology, it is possible to modulate tube current during the cardiac cycle, which allows evaluation of multiple phases from systolic to diastolic phase (e.g., 30–70%) at acceptable radiation dose.

## Conclusion

In conclusion, this study investigated the dynamic behavior of coronary artery geometry during the cardiac cycle using a four-dimensional, non-invasive imaging method. The results of this study indicate that hypothesis 1 is accepted as coronary artery geometry changes significantly between ES and ED phases. However, this study could not prove that dynamic change of vessel geometry through the cardiac cycle are related to the presence of CAD which led to the rejection of hypothesis 2, in contrast to prior findings using a single cardiac phase. Nevertheless, the non-invasive nature of this study allows further investigation of the relationship between the dynamic behavior of coronary arteries and CAD with larger and more balanced subject population in order to study potential biomechanical mechanisms underlying CAD development.
